# Supplementary material for: ProteinShader: illustrative rendering of macromolecules
Source: BMC Struct Biol. 2009 Mar 30;9:19. doi: 10.1186/1472-6807-9-19 (PMC2672931; doi:10.1186/1472-6807-9-19)
Supplement: Additional file 1 — ProteinShader program without source code. This compressed file contains the complete ProteinShader program including associated libraries, but no source code. A README.txt file gives an overview of the ProteinShader distribution, and the index.html file in the help subdirectory has directions on getting started with the program as well as a set of tutorials. [file 1472-6807-9-19-S1.zip › ProteinShader-beta-0_9_4-binary/help/getting-started.html]

ProteinShader: Getting Started


# Getting Started with the ProteinShader Program


♦ Home


♦ Download


♦ Getting Started

Choose a Link:

Installation

Running

Troubleshooting

PDB Files


♦ Tutorials

Choose a Link:

List of Proteins

Style Options

Saving Images


♦ General Controls

Choose a Link:

Menu Bar

Keyboard Shortcuts

Mouse Motion

Motion Panel

Antialiasing


♦ Cartoon Controls

Choose a Link:

Control Panels

Selection Panel

Radio Buttons

Decorations Panel

Adding Textures

Color Panel

Visibility Panel

Side Chains Panel


♦ Atom Controls

Choose a Link:

Control Panels

Selection Panel

Radio Buttons

Color Panel

Visibility Panel

Scale Panel

Tiling Panel


♦ Developers

Choose a Link:

Compiling

Running

Code Docs

Developer Links


## Installation

- The ProteinShader program is currently distributed as a zip file. If a
  computer already has Java 1.5 or higher, and the graphics card supports
  at least OpenGL 2.0, installation should involve nothing more than
  decompressing the zip file.

- ### System Requirements: Java 1.5 and OpenGL 2.0

  To make it platform independent, the ProteinShader program is written
  primarily in Java, and has been tested on computers using the Linux,
  Macinosh OS X, and Windows XP operating systems. Java 1.5 or later is
  required. One way to test what version of Java is installed is to
  open a terminal window (Terminal on Macintosh OS X / Linux or Command
  Prompt on Windows XP) and type "java -version" on the command
  line.

  On Macintosh OS X, Java 1.5 is probably already installed, or
  can be obtained by using the built-in Macintosh Software Update tool
  to automatically download and install it. For Windows XP and Linux,
  Java 1.5 can be obtained from the
  Sun Microsystems web site.
  The Sun web site often refers to Java 1.5 as Java 2 Standard Edition
  5.0. Similarly, Java 1.6, which has been out for a while now, is often
  referred to as Java 2 Standard Edition 6.0. For developers who wish to
  modify and recompile the source code, the Java SE Development Kit (JDK)
  5.0 or higher is needed. For end-users who only want to run the
  program, the Java Runtime Environment (JRE) 5.0 or higher is all
  that is needed.

  When the ProteinShader program first starts up, it will check that the
  graphics card supports OpenGL 2.0. If an earlier version of OpenGL is
  found, a dialog box will open up to inform the user of the version
  number problem, and the program will exit. OpenGL 2.0 was finalized on
  October 22, 2004, and is supported by most good quality graphics cards
  produced since then.

  OpenGL 2.0 or later is required because ProteinShader includes
  several small programs, known as vertex and fragment shaders, that are
  written using the OpenGL Shading Language. These shaders perform the
  custom lighting calculations and texture mapping that allow
  illustrative rendering effects such as pen-and-ink style drawings.
  Full support for OpenGL Shading Language became standardized as of
  OpenGL 2.0.

- ### Unpacking the Zip File

  Assuming that Java 1.5 (or higher) and OpenGL 2.0 (or higher) are
  already available, installing the ProteinShader program requires
  nothing more than decompressing the ProteinShader-XX.zip file (where
  XX is a version number). On most operating systems, simply
  double-clicking on a zip file will call on a decompression tool.
  Depending on the decompression tool, it may be necessary to select
  something like "Extract All" from a menu. If for some reason
  a zip decompression program is not found, the Java jar tool can be
  used to decompress a zip file. Open a Terminal window on Macintosh
  OS X or Linux (Command Prompt on Windows XP), cd into the directory
  the zip file is in, and type "jar -xf ProteinShader-XX.zip"
  on the command line.

  The location of the decompressed `ProteinShader-XX` directory
  should not matter, but the internal directory structure should not be
  changed. The ProteinShader program is a runnable JAR file,
  ProteinShader.jar, located in the `bin` subdirectory. The
  compiled Java code in ProteinShader.jar knows the relative location of
  most of the subdirectories of the `ProteinShader-XX`
  directory (`data`, `docs`, `fonts`,
  `help`, `images`, `shaders`,
  `textures`) because it needs to access those directories at
  run time. Also, inside the `bin` directory where
  ProteinShader.jar is found there are several dynamic libraries
  ('.so' files for Linux, '.jnilib' files for Macintosh OS X, and '.dll'
  files for Windows XP) that need to remain at that location.


## Running

- The ProteinShader program has been tested on Windows XP, Macintosh OS X,
  and Linux.

- #### Windows XP

  Double-click on the ProteinShader.jar file in the
  `bin` directory. It may take several seconds or
  longer to start the Java Virtual Machine if it has not been used
  recently, so be patient. For convenience, an alias to the
  ProteinShader.jar file could be created and placed on the
  desktop.

  On most Windows machines, double-clicking on the
  ProteinShader.jar file should work fine. However, if the
  program has difficulty finding the dynamic libraries that it
  needs on Windows (jogl.dll, jogl\_awt.dll, and gluegen-rt.dll in
  the `bin` directory), it might be necessary to start
  the program by using the run.bat batch file located in the
  directory right above the `bin` directory. The
  run.bat file can be used by either double-clicking on it or by
  opening a Command Prompt window and typing "run.bat" at
  the command line.

  The run.bat batch file contains the code

   `cd .\bin   
  java -Djava.library.path=. -jar ProteinShader.jar   
  cd .\..   
  pause` 

  If the batch file was started by double-clicking on it, the
  pause command at the end will cause the Command Prompt window
  to stay open even if something goes wrong, and can be closed
  by selecting the window and pressing any key, or by just
  clicking the close box at the upper right of the Comand Prompt
  window. On most Windows machines, the default search path for
  dynamic libraries usually includes the current directory
  (the directory the Java Virtual Machine started up in), so the
  "-Djava.library.path=." argument is probably
  unnecessary, and has been included only to deal with the rare
  case that someone may have set the Java library path to
  something else.

- #### Macintosh OS X

  The simplest way to start the program on Macintosh OS X is to
  go into the `bin` directory and double-click on the
  ProteinShader.jar file. It may take several seconds or longer
  to start the Java Virtual Machine if it has not been used
  recently, so be patient. For convenience, an alias to the
  ProteinShader.jar file could be added to the desktop or dock.

  On most Macintosh OS X machines, the program should have no
  problem finding the dynamic libraries that it needs
  (libjogl.jnilib, libjogl\_awt.jnilib, and libgluegen-rt.jnilib
  in the `bin` directory). However, if a problem
  occurs, it may be necessary to start the program by opening a
  Terminal window, changing into the top level directory of the
  ProteinShader-beta distribution (the directory where
  `bin` can be seen), and typing "sh run.sh".

  The run.sh shell script contains the code

   `cd ./bin   
  java -Djava.library.path=. -jar ProteinShader.jar` 

  The "-Djava.library.path=." part of the command
  explicitly tells the Java Virtual Machine where to find the
  dynamic libraries that it will need.

- #### Linux

  To start the program on Linux, open the ProteinShader directory
  and double-click on the run.sh shell script. It may be
  necessary to first set the permissions on the run.sh script
  to include execute, which can be done by right clicking on
  run.sh with a mouse to open a properties dialog box, or by
  typing "chmod 700 run.sh" on the command line.
  The script could of course also be run by typing "sh
  run.sh" on the command line. The script contains the code

   `cd ./bin   
  java -Djava.library.path=. -jar ProteinShader.jar` 

  The "-Djava.library.path=." part of the command tells
  the Java Virtual Machine where to find the dynamic libraries
  that it will need (libjogl.so, libjogl\_awt.so, and
  libgluegen-rt.so in the `bin` directory). On my
  Linux installation (Fedora Core 5), double-clicking on the
  ProteinShader.jar file in the `bin` directory fails
  to start the program because the dynamic libraries are not
  found, so on at least some Linux installations the run.sh
  script is necessary.

  On machines more than a year or two old, it may take several
  seconds or longer to start the Java Virtual Machine if it has
  not been used recently, so be patient.


## Troubleshooting

- This section addresses the two most likely problems that could prevent
  the ProteinShader program from starting. The first potential problem
  is that even though Java 1.5 is installed, it cannot be found because
  the PATH environment variable is not set correctly. The second
  potential problem is an UnsatisfiedLinkError that will occur if the
  Java Virtual Machine cannot find the dynamic libraries needed for
  JOGL (Java Bindings for OpenGL).

- #### PATH environment variable

  Even if Java 1.5 is installed, the operating system could have a
  problem finding it. In a Terminal window on Linux or Macintosh
  OS X (or a Command Prompt window on Windows XP), type "java
  -version" on the command line. If Java 1.5 is installed,
  but a "command not found" message results (or an earlier
  version number is reported), then the PATH environment variable
  does not include the path to the Java 1.5 installation.

  For Linux or Macintosh OS X, setting the PATH environment
  variable could be done in a profile or login file that is read
  whenever a Terminal window is opened. The name of the file and
  the command that needs to be added will vary depending on what
  shell is in use and possibly other details of the operating
  system installation, so instead of modifying the PATH
  environment variable, a simpler solution might be to just add
  the path to the run.sh script. For example, if the Java 1.5
  java command was in the directory
   `/usr/java/jdk1.5.0_07/bin` ,
  then the run.sh script shown further above would need to be
  changed to

   `cd ./bin   
  /usr/java/jdk1.5.0_07/bin/java -Djava.library.path=. -jar
  ProteinShader.jar` 

  For Windows XP, if the Java 1.5 java command was in the directory
   `c:\Program Files\Java\jdk1.5.0_07\bin` ,
  the run.bat script could be changed to

   `cd .\bin   
  "c:\Program Files\Java\jdk1.5.0_07\bin\java"
  -Djava.library.path=. -jar ProteinShader.jar   
  cd .\..   
  pause` 

  The double quotes are needed because of the blank space in the
  path. The directory separator can be either the forward slash
  or the back slash on Windows XP (although only the forward
  slash works on Linux or Macintosh OS X).

  An alternative would be to set the Path environment variable in
  the Windows XP System Properties box. This box can be opened by
  clicking on Start and selecting Control Panel. When the
  Control Panel opens, double-click System. Select the Advanced
  tab and then click the button for Environment Variables. Under
  System Variables, scroll down until Path is seen. Select Path
  and click the edit button. The path
  "c:\Program Files\Java\jdk1.5.0\_07\bin\java;"
  (but without the quotes now) should be added to the very
  beginning of the Path. The semicolon at the end is the path
  separator (there will already be several search paths specified
  in the Path environment variable).

- #### The dreaded UnsatisfiedLinkError

  The ProteinShader program has several dynamic libraries that are
  needed by Java Bindings for OpenGL (JOGL). These dynamic
  libraries ('.so' files for Linux, '.jnilib' files for Macintosh
  OS X, and '.dll' files for Windows XP) have been placed in the
  `bin` directory at the same level as
  ProteinShader.jar in order to make it easy for the JAR file to
  find them.

  On most machines that are set up to allow a runnable JAR file
  to be started by double-clicking, the default java.library.path
  will include the current directory (the directory the JAR file
  is in), so the dynamic libraries should be found automatically.
  However, if the current directory is not in the
  java.library.path, an UnsatisfiedLinkError will occur, and the
  program will need to be started by a script that includes the
  argument "-Djava.library.path=." after the java command.

  The run.sh script discussed further above (for Macintosh OS X
  or Linux) includes this argument because some Linux
  installations (or at least my own Linux Fedora Core 5
  installation) apparently do not include the current directory
  in java.library.path. I have not seen this problem on Windows
  XP, but included the "-Djava.library.path=." in the
  run.bat file as a precaution in case someone has modified the
  Java installation to something other than the default.

  The version of JOGL used by ProteinShader is JSR-231 version
  1.1.1 (May 22, 2008), and the jogl.jar and gluegen-rt.jar
  libraries are in the `lib` subdirectory. The
  associated dynamic libraries were downloaded as zip files from
  https://jogl.dev.java.net:
    
    
  jogl-1.1.1-linux-i586.zip (binaries for Linux/x86)
    
  jogl-1.1.1-macosx-universal.zip (binaries for Mac OS X 10.4+)
    
  jogl-1.1.1-windows-i586.zip (binaries for Windows/x86)

  Macintosh OS X (Intel), Windows XP/x86, and Linux/x86 are the
  only platforms the ProteinShader has been tested on, so those
  are the only dynamic libraries included in the `bin`
  directory. The following dynamic libraries for Solaris and
  AMD64 machines are available at
  https://jogl.dev.java.net,
  but have not been tested with the ProteinShader program:
    
    
  jogl-1.1.1-linux-amd64.zip   
  jogl-1.1.1-windows-amd64.zip   
  jogl-1.1.1-solaris-amd64.zip   
  jogl-1.1.1-solaris-i586.zip   
  jogl-1.1.1-solaris-sparc.zip   
  jogl-1.1.1-solaris-sparcv9.zip


## Protein Data Bank Files

- ### Opening a PDB File

  When "Open..." is selected from the File menu at the top left of the
  ProteinShader GUI, a dialog box will open to the
  `data` directory, which should already have several
  PDB structure files. Currently, the ProteinShader program only reads
  PDB formatted text files, which usually have a filename consisting of
  the PDB ID followed by a ".pdb" extension. Support for XML and
  mmCIF will likely be added at some future time. Also, the first version
  of the ProteinShader only reads protein structures. Support for DNA
  and RNA will be added at a later time.

- ### Obtaining PDB Files

  To obtain additional PDB structure files, point a web brower at
  www.rcsb.org. The PDB web
  site has a search engine that allows a user to quickly find a structure
  entry by keywords, author, or PDB ID. On the left side of a PDB
  structure entry page, there will be a Download Files menu near the
  top left of the page. Select "PDB text" to download a file,
  or select "PDB.gz" to dowload a compressed version. For
  convenience, the file should be placed in the `data`
  directory because that is where the file chooser box for the
  ProteinShader program will open to by default.


---

|  |  |  |  |
| --- | --- | --- | --- |
| Hosted by |  |  |  |

Webmaster: Joe Weber,
joe.weber AT alumni.duke.edu
  
Web site last updated: December 30th, 2008
  
© 2007–2008 Joseph R. Weber
